# Association of high-density lipoprotein cholesterol with reduced intracranial haemorrhage and favourable functional outcome after thrombectomy for ischaemic stroke: a propensity-matched analysis

**DOI:** 10.1186/s42466-025-00373-4

**Published:** 2025-03-10

**Authors:** Annahita Sedghi, Sonja Schreckenbauer, Daniel P. O. Kaiser, Ani Cuberi, Witold H. Polanski, Martin Arndt, Kristian Barlinn, Volker Puetz, Timo Siepmann

**Affiliations:** 1 Dresden Neurovascular Center, Department of Neurology, Medical Faculty and University Hospital Carl Gustav Carus, TUD Dresden University of Technology, Fetscherstraße 74, 01307 Dresden, Germany; 2 Dresden Neurovascular Center, Institute of Neuroradiology, Medical Faculty and University Hospital Carl Gustav Carus, TUD Dresden University of Technology, Fetscherstraße 74, 01307 Dresden, Germany; 3 Institute of Radiology, Medical Faculty and University Hospital Carl Gustav Carus, TUD Dresden University of Technology, Fetscherstraße 74, 01307 Dresden, Germany

**Keywords:** Ischaemic stroke, High density lipoprotein, Endovascular therapy, Thrombectomy, Reperfusion injury, Bleeding, Intracranial haemorrhage, Functional outcome

## Abstract

**Background:**

Animal studies suggest that high-density lipoprotein cholesterol (HDL-C) attenuates reperfusion injury. We aimed to assess whether higher serum HDL-C levels modulate the risk of intracranial haemorrhage (ICH) after thrombectomy in human stroke survivors.

**Methods:**

We included consecutive patients from our prospective anterior circulation large vessel occlusion (acLVO) registry who underwent thrombectomy between 01/2017 and 01/2023 at the tertiary stroke centre of the University Hospital Carl Gustav Carus in Dresden, Germany in a propensity score-matched analysis. We assessed the association between serum HDL-C levels and post-interventional ICH as well as 90-day functional outcome quantified by the modified Rankin Scale (mRS). For sensitivity analysis, we used multivariable lasso logistic regression. Analyses were adjusted for demographics, cardiovascular risk profiles, stroke characteristics, and procedural times.

**Results:**

Of 1702 patients screened, 807 (420 women, median age 77 years [66–84, IQR]) were included. Post-interventional ICH reduced the probability of a favourable functional outcome (90-day mRS 0–2) by 14.8% (ß = 0.15; 95% CI [0.06;0.24]; *p* = 0.001. An HDL-C level above the median (1.15 mmol/L) decreased the probability of ICH by 13.6% (ß = − 0.14; 95CI% [− 0.22; − 0.05]; *p* = 0.002) and increased the probability of favourable functional outcome by 13.2% (ß = − 0.13; 95CI% [− 0.22; − 0.05]; *p* = 0.003). In sensitivity analyses, higher HDL-C levels were independently associated with lower odds of ICH (adjusted OR 0.62; 95% CI [0.43;0.88]; *p* = 0.008) and higher odds of favourable functional outcome (adjusted OR 0.60; 95% CI [0.40; 0.90]; *p* = 0.015).

**Conclusions:**

In patients undergoing thrombectomy for acLVO, higher HDL-C levels were associated with a reduced probability of post-interventional ICH and a favourable functional outcome. These observations could not be explained by conventional vascular risk profiles.

**Supplementary Information:**

The online version contains supplementary material available at 10.1186/s42466-025-00373-4.

## Background

Thrombectomy for anterior circulation large vessel occlusion (acLVO) has revolutionised stroke care ever since randomised clinical trials published in 2015 as well as their meta-analytic synthesis have confirmed the efficacy and safety of the treatment; an observation that has more recently been reproduced in an extended time window of up to 24 h after stroke onset, large core infarcts and basilar artery occlusion [1, 2]. Observational studies and sub-analyses of randomized trials have shown that the beneficial effects of thrombectomy may be attenuated by reperfusion injury [3]. Abrupt reperfusion of ischaemic brain tissue can lead to local inflammation and oxidative stress, endothelial damage, and oedema, resulting in a breakdown of the blood–brain barrier with possible haemorrhage. This complication has been reported after carotid endarterectomy and stenting and, more recently, after thrombectomy [4–6]. In brain tissue affected by reperfusion injury, haemorrhage may manifest as haemorrhagic transformation, parenchymal or subarachnoid haemorrhage [7–9]. Radiologically confirmed haemorrhagic reperfusion injury occurs in up to one third of patients after thrombectomy with partial or complete reperfusion, worsening functional outcome [7]. To date, the optimal strategy to prevent this complication is unknown. There remains an urgent need for therapeutic targets to protect brain tissue from reperfusion injury after thrombectomy.

Animal studies have shown that high-density lipoprotein cholesterol (HDL-C)-based treatment exerts direct vasculoprotective and neuroprotective effects [10], preserves the integrity of the blood–brain barrier after transient intraluminal occlusion of the middle cerebral artery [12], and reduces the risk of bleeding after treatment with tissue plasminogen activator [13]. To our knowledge, the effects of HDL-C on cerebral reperfusion injury after thrombectomy in humans are unknown.

## Methods

### Aim

We aimed to test the hypothesis that patients with higher serum HDL-C levels undergoing thrombectomy for acLVO would have a lower risk of intracranial haemorrhage (ICH) after the intervention than those with lower HDL-C levels.

### Design, setting and patients

We included all adult acLVO patients from our prospective endovascular treatment registry who underwent thrombectomy at the tertiary stroke centre of the University Hospital Carl Gustav Carus in Dresden, Germany between 01/2017 and 01/2023 in a retrospective cohort study. This study report conforms to the Strengthening the Reporting of Observational Studies in Epidemiology (STROBE) statement [14]. The *STROBE checklist* is provided in Additional file 1. We excluded patients with an incomplete or missing lipid panel, missing follow-up cranial imaging, unknown symptom onset or time of last seen well, unknown baseline National Institute of Health Stroke Scale (NIHSS) score or missing modified Rankin Scale (mRS) score at 90 days. Definitions of patient characteristics are available in Additional file 2.

### Prospective thrombectomy registry

Our registry of thrombectomy-eligible patients includes detailed data on demographic characteristics, cardiovascular risk profiles, premorbid conditions, chronic comorbidities, medications, stroke aetiology as classified by the Trial of Org 10,172 in Acute Stroke Treatment (TOAST), modified treatment in cerebral infarction (mTICI) score, neurological deficits as quantified by the National Institute of Health Stroke Scale (NIHSS), and functional outcome as assessed by the modified Rankin Scale (mRS) at admission, discharge, and 90 days. Fasting serum lipid profiles were obtained according to institutional protocol within the first 72 h after admission to the stroke unit. Details of the registry are provided in Additional file 3, and the details of the collection of baseline and outcome parameters are provided in Additional file 4, respectively.

### Assessment of imaging and clinical outcomes

We considered post-interventional ICH to be present if at least one of the key radiological features of haemorrhagic reperfusion injury was evident on follow-up cranial computed tomography or cranial magnetic resonance imaging within 24 h of thrombectomy. These key features included any haemorrhagic transformation, defined as Heidelberg Bleeding Classification categories HI1 and HI2, and any parenchymal (PH1 and PH2) or subarachnoid (class 3c) haemorrhage involving the ischaemic brain region, as previously proposed [9, 15]. All brain scans were evaluated by board-certified neuroradiologists. Successful recanalisation was defined.

as an mTICI score of 2b or greater.

Functional outcome was assessed by telephone interview 90 days after thrombectomy. Favourable functional outcome was defined as a modified Rankin Scale (mRS) score of 0–2 and poor functional outcome as an mRS score ≥ 3 at the time of this follow-up. Neurological deficits were assessed on admission and discharge using the National Institutes of Health Stroke Scale (NIHSS). Symptomatic ICH was defined as evidence of ICH on cranial imaging prompted by neurological deterioration.

### Statistical analysis

Independent continuous variables were tested for normality using descriptive and analytical criteria (Shapiro–Wilk test). Differences between groups in demographic, clinical, imaging, and procedural characteristics were assessed using Fisher’s exact test for binary data and Kruskal Wallis test for categorical or non-normally distributed continuous data, as appropriate.

Propensity score matching was performed for the main analysis. Patients were divided into two groups based on their serum HDL-C level, with the cut-off value set at the median HDL-C level of the entire study population. Accordingly, low HDL-C was defined as an HDL-C level < 1.15 mmol/L and high HDL-C ≥ 1.15 mmol/L. Covariates were selected by multivariable logistic regression including clinically relevant covariates, namely age, sex, premorbid dependency, chronic diseases that may affect functional independence, baseline NIHSS score, Alberta Stroke Program Early CT Score (ASPECTS), occlusion site, concomitant extracranial carotid occlusion, mTICI score, emergency carotid stenting, thrombectomy, onset-to-recanalization time, arterial hypertension, HbA1c (%) and serum level of low-density lipoprotein cholesterol (LDL-C) (mg/dl). Propensity score matching was performed for all covariates that showed a statistically significant association with the grouping variable and the outcome on this regression model. Propensity scores were generated using logistic regression. One-to-one nearest neighbour matching with calliper adjustment was applied. The maximum allowed difference in propensity scores for matching (calliper value) was aimed at ≤ 0.2 (fraction of the standard deviation of the logit of the propensity score) to ensure high quality matching. Visual and analytical comparisons were performed to assess the quality of the matches. We calculated the standardized difference of covariate means and the distribution of covariates between groups to assess the overall balance of covariates between two groups. We aimed for a standardized difference of 10% (0 ± 0.1) and a variance ratio of 1 ± 0.2 to ensure a good balance. The average treatment effect was estimated.

In the sensitivity analysis, we performed multivariable lasso regression to assess the association between serum HDL-C levels and imaging indices of post-interventional ICH and favourable functional outcome, as well as early NIHSS score at discharge, adjusting for clinically relevant covariates. Residuals were tested for normality. We performed a shift analysis to compare the low and high serum HDL-C groups. We used ordered multivariate logistic regression to assess the relationship between HDL-C and functional outcome as defined by mRS score from 0 to 6. Available case analysis was carried out. Statistical significance was set at *p* < 0.05. All analyses were performed using Stata® (StataCorp. 2021. Stata Statistical Software: Release 17. College Station, TX: StataCorp LLC).

## Results

### Study population

Of 1702 patients with cerebral large vessel occlusion assessed for eligibility, we included 807 patients treated with thrombectomy for acLVO (420 females, median age 77 years [interquartile range, IQR 66–84]); median baseline NIHSS 15 [IQR 10–19]; median baseline mRS 5 [IQR 4–5]; median ASPECTS 7 [IQR 6–9], of whom 403 (49.7%) received preceding intravenous thrombolysis (IVT). Recombinant tissue plasminogen activator was used as the lytic agent in all cases of IVT.

**Fig. 1:**
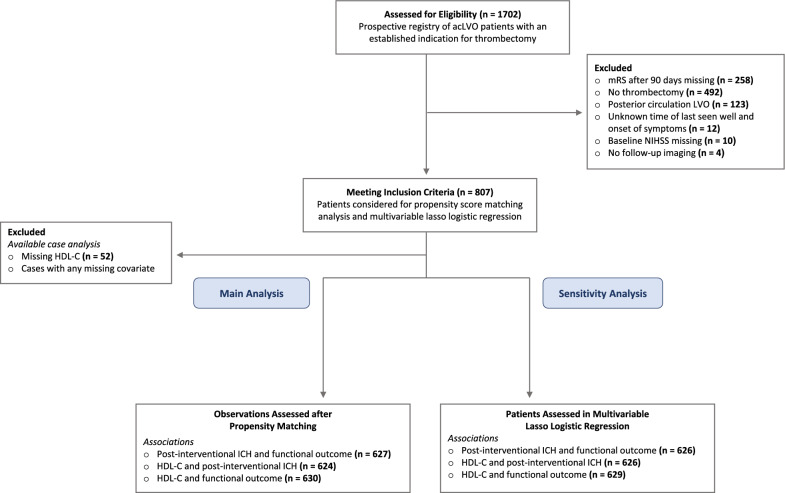
Study flowchart *Legend:* Study flowchart illustrating the screening and selection process of patients for inclusion in the main and sensitivity analyses of the study

Post-interventional ICH occured in 192 (49.7%) of 386 patients with high serum HDL-C and in 123 (33.3%) of 369 patients with low serum HDL-C. The median mRS score at 90 days was 3 [IQR 1–4] in the high serum HDL-C group and 4 [IQR 2–6] in the low serum HDL-C group. The median NIHSS score at 90 days was 5 [IQR 1–14] in the high serum HDL-C group and 9 [IQR 2–19] in the low serum HDL-C group. Demographic and clinical characteristics, vascular risk profiles and imaging characteristics are detailed in Table 1. Subject selection and reasons for exclusion are shown in the study flowchart (Fig. 1). The number of missing registry data was low. Details are provided in Additional file 5. Study outcome measures are shown in Table 2.

**Table 1:** Demographic and baseline characteristics

	Patients receiving thrombectomy (n = 807)	Low HDL-C (< 1.15mmol/L) (n = 386)	High HDL-C (≥ 1.15 mmol/L) (n = 369)	*p*-value
*Demographic Characteristics*				
Age, years (median [IQR])	77 [66, 84]	75 [64, 83]	78 [69, 84]	0.018
Sex, female (n, %)	420, 52.00	155, 40.16	235, 63.69	0.000
*Premorbid condition*				0.885
Independent (n, %)	587, 72.73	283, 73.32	269, 72.90	
Needs assistance (n, %)	224, 27.76	103, 26.68	100, 27.10	
*Chronic disease (n, %)*				0.104
No organ system	501, 62.08	230, 59.59	238, 64.50	
1 Organ system	220, 27.26	107, 27.72	100, 27.10	
2 Organ systems	80, 9.91	42, 10.88	30, 8.13	
3 Organ systems	9, 1.12	7, 1.81	1, 0.27	
*Cardiovascular Risk Factors*				
Arterial hypertension (n, %)	720, 89.22	347, 89.90	323, 87.53	0.164
Diabetes mellitus (n, %)	232, 28.75	137, 35.49	80, 21.68	0.000
HbA1c, % (median [IQR])	5.8 [5.4, 6.3]	5.9 [5.5, 6.4]	5.7 [5.4, 6.1]	0.000
LDL, mg/dl (median [IQR])	2.28 [1.71, 2.99]	2.17 [1.64, 2.88]	2.41 [1.81, 3.04]	0.004
HDL, mg/dl (median [IQR])	1.15 [0.93, 1.43]	0.93 [0.78, 1.05]	1.41 [1.27, 1.58]	
*Preventive Pharmacological Treatment*				
Statin (n, %)	262, 32.47	136, 35.23	114, 30.89	0.112
Antiplatelet therapy (n, %)	205, 25.40	105, 27.20	90, 24.39	0.194
Dual Antiplatelet therapy (n, %)	7, 0.87	3, 0.78	3, 0.81	
Aspirin monotherapy (n, %)	191, 23.67	96, 24.87	87, 23.58	
Clopidogrel monotherapy (n, %)	7, 0.87	6, 1.55	0,0	
*Stroke Characteristics*				
Wake-up	163, 20.20	72, 18.65	80, 21.68	0.179
NIHSS at baseline (median, [IQR])	15 [10, 19]	15 [11, 19]	15 [10, 18]	0.043
mRS at baseline (median [IQR])	5 [4, 5]	5 [4, 5]	5 [4, 5]	0.088
ASPECTS (median [IQR])	7 [6, 9]	7 [6, 9]	8 [6, 9]	0.401
Occlusion side, left (n, %)	410, 50.81	205, 53.11	172, 46.61	0.068
Occlusion site (n, %)				0.008
ICA intracranial	9, 1.12	6, 1.55	3, 0.81	
Carotid L or isolated M1	662, 82.03	326, 84.46	290, 78.59	
M1/2-junction or isolated M2	139, 17.14	53, 13.73	76, 20.60	
Carotid T	80, 9.91	39, 10.10	31, 8,40	
Tandem	105, 13.01	62, 16.06	33, .8.94	
Leptomeningeal collaterals on DSA (n, %)	673, 83.40	312, 80.83	313, 84.82	0.052
TOAST Classification (n, %)				0.001
Large artery atherosclerosis	150, 18.59	87, 22.54	53, 14.36	
Cardioembolism	474, 58.74	220, 56.99	220, 59.62	
Small vessel occlusion	0, 0.00	0, 0.00	0, 0.00	
Stroke of other determined aetiology	23, 2.85	9, 2.33	10, 2.71	
Stroke of undetermined aetiology	161, 19.95	67, 17.36	86, 23.30	
*Interventions (n, %)*				
Intravenous thrombolysis with rtPA	403, 49.94	194, 50.26	187, 50.68	0.498
Drip and ship	529, 65.55	265, 68.65	227, 61.52	0.024
Carotid stent (emergency)	87, 10.78	54, 13.99	27, 7.32	0.002
Sedative Regimen (n, %)				**0**.004
Conscious sedation	232, 28.75	95, 24.61	123, 33.33	
General anaesthesia	562, 69.64	284, 73.58	238, 64.50	
*Procedural Times, min (median [IQR])*				
Onset unclear (n, %)	123, 15.24	53, 13.73	58, 15.72	0.397
Onset-to-needle	105 [80, 135]	109.5 [85, 138]	105 [79, 134]	0.470
Onset-to-groin	240 [173, 298]	242.2 [174, 298]	235 [171, 300]	0.967
Onset-to-recanalization	302 [234, 364]	301 [239, 367]	297 [228, 360]	0.318

Abbreviations: ASPECTS, Alberta Stroke Program Early CT score ICA; carotid L occlusion, occlusion of distal intracranial ICA and proximal M1 segment; carotid T occlusion, combined distal intracranial ICA occlusion and ipsilateral proximal M1 and A1 occlusion; DSA, digital subtraction angiography; HbA1C, haemoglobin A1c; internal carotid artery; HDL-C, high-density lipoprotein cholesterol; IVT, intravenous thrombolysis; IQR, interquartile range; LDL-C, low-density lipoprotein cholesterol; mRS, modified Rankin scale; MT, mechanical thrombectomy; SD standard deviation; NIHSS, National Institutes of Health Stroke Scale; tandem occlusion, extracranial ICA occlusion or high-grade stenosis (at least 70% stenosis on the North American Symptomatic Carotid Endarterectomy Trial scale, NASCET) preceding ipsilateral acLVO. The high HDL-C and low HDL-C groups only include cases with available serum HDL-C concentrations, as detailed in Fig. 1

**Table 2:** Outcomes

	Patients receiving thrombectomy (n = 807)	Low HDL-C (< 1.15mmol/L) (n = 386)	High HDL-C (≥ 1.15 mmol/L) (n = 369)	*p*-value
*Procedural Outcomes (n, %)*				0.887
Thrombectomy frustrate	89, 11.02	41, 10.62	39, 10.57	
mTICI (n, %)				
0	70, 8.67	30, 7.77	32, 8.67	
1	8, 0.99	4, 1.04	4, 1.08	
2a	40, 4.96	25, 6.48	13, 3.52	
2b	276, 34.20	129, 33.42	131, 35.50	
2c, 3	417, 51.67	198, 51.30	189, 51.22	
*Post-interventional ICH (n, %)*				0.000
Any post-interventional ICH	337, 41.76	192, 49.74	123, 33.33	
Haemorrhagic transformation (HI1, HI2)	211, 26.15	122, 31.61	78, 21.14	
Intracranial bleeding (PH1, PH2)	85, 10.53	47, 12.18	33, 8.94	
Subarachnoid haemorrhage (Class 3c)	118, 14.62	68, 17.62	41, 11.11	
Symptomatic haemorrhage	17, 2.11	10, 2.59	5, 1.36	
*Clinical Outcomes (n, %)*				
NIHSS at discharge (median, [IQR])	7 [2, 17]	9 [2, 19]	5, [1, 14]	0.001
mRS at discharge (median [IQR])	4 [2, 5]	4 [2, 5]	3 [2, 5]	0.002
mRS at 90 days (median [IQR])	3 [2, 6]	4 [2, 6]	3 [1, 4]	0.000
mRS (0–2) at 90 days (n, %)	290, 35.94	121, 31.35	158, 47.82	0.002

Class 3c, subarachnoid haemorrhage according to Heidelberg Bleeding Classification; HDL-C, high-density lipoprotein cholesterol; HI, haemorrhagic infarction according to Heidelberg bleeding classification; ICH, intracranial haemorrhage; IQR, interquartile range; mTICI, modified treatment in cerebral infarction score; NIHSS, National Institutes of Health Stroke Scale; mRS, modified Rankin scale; PH, parenchymal haematoma according to Heidelberg Bleeding Classification

### Association of post-interventional ICH with functional outcome

**Fig. 2:**
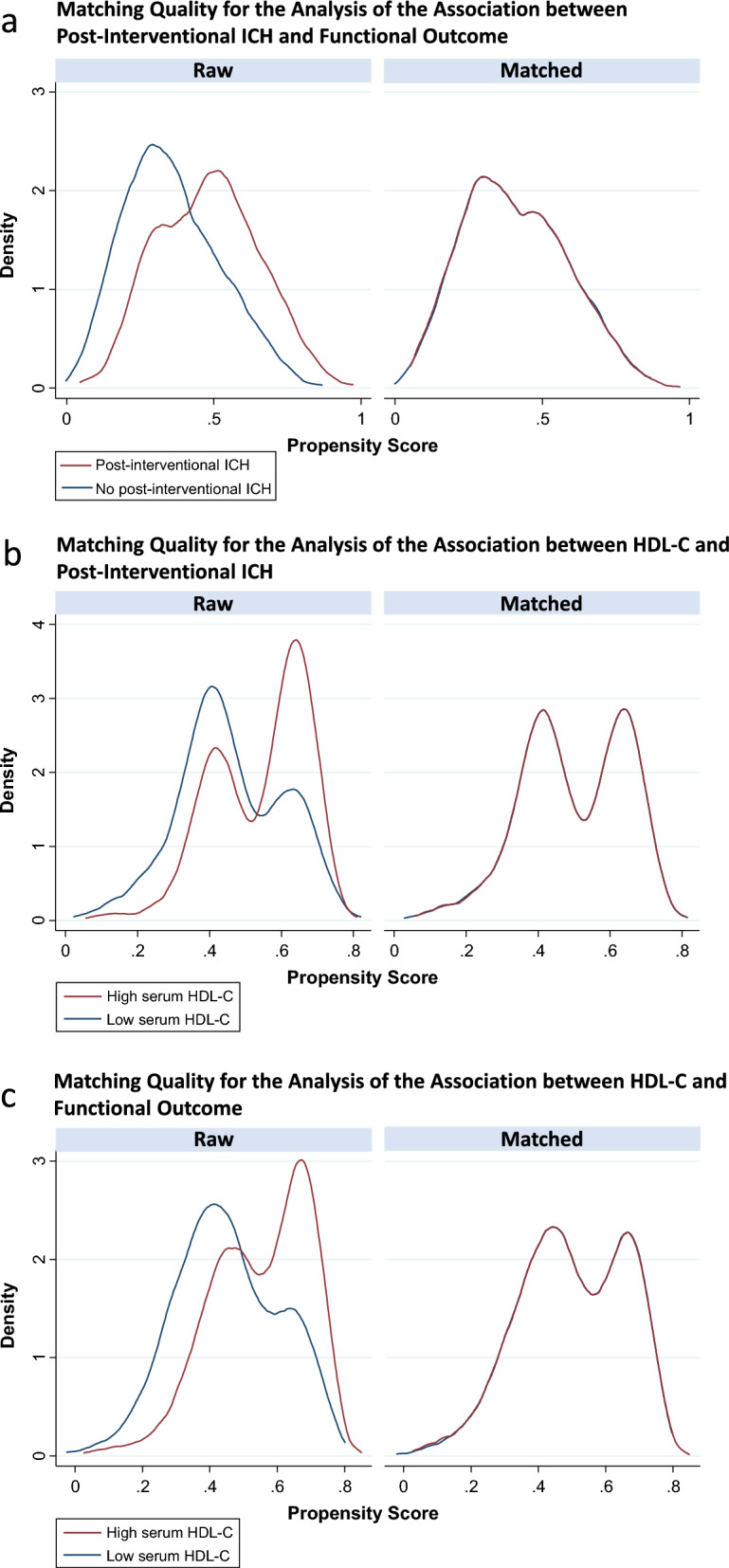
Matching quality *Legend:* Propensity score density plots for the analyses of A) the association between post-interventional ICH and functional outcome; B) the association between HDL-C and post-interventional ICH; and C) the association between HDL-C and functional outcome. The quality of the matching was good in all cases

The presence of post-interventional ICH on brain imaging was associated with a 14.8% increase in the probability of poor functional outcome (ß = 0.15; 95CI% [0.06; 0.24]; *p* = 0.001). The quality of the matching was good, as shown in Fig. 2A and Additional file 6. This observation was confirmed in the sensitivity analysis, where post-interventional ICH was independently associated with higher odds of poor functional outcome (adjusted OR 2.69; 95% CI [1.75; 4.14]; *p* = 0.000).

### Association of serum HDL-C levels and post-interventional ICH

A high serum HDL-C level was associated with a 13.6% reduction in the probability of post-interventional ICH (ß = −0–14; 95CI% [−0.22; 0.05]; *p* = 0.002). The quality of the matching was good, as shown in Fig. 2B and Additional file 7. This observation was confirmed in the sensitivity analysis, where a high serum HDL-C level was independently associated with lower odds of post-interventional ICH (adjusted OR 0.62; 95% CI [0.43; 0.88]; *p* = 0.008).

### Association of HDL-C levels and functional outcome

**Fig. 3:**
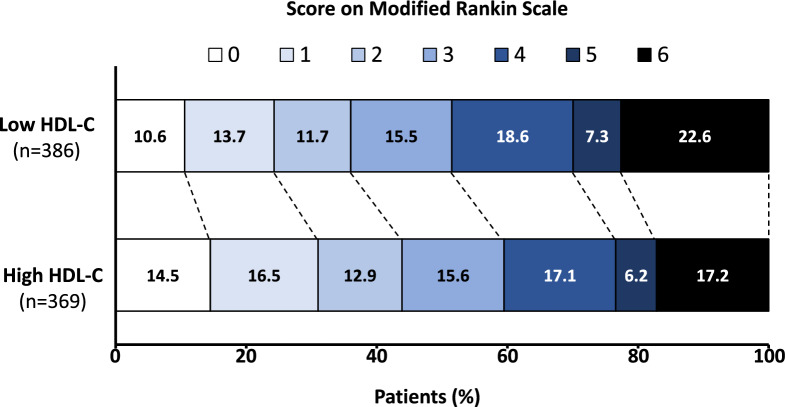
Shift analysis *Legend:* Modified Rankin scale scores at 90 days are shown, derived from ordered logistic regression with adjustment for age, sex, cardiovascular risk profile, intravenous thrombolysis, baseline NIHSS, premorbid condition, chronic disease, onset-to-recanalisation time, occlusion site, carotid stenting, modified treatment in cerebral infarction (mTICI) score, Alberta stroke programme early CT score (ASPECTS)

A high serum HDL-C level was associated with a 13.2% increase in the probability of achieving a favourable functional outcome (ß = − 0.13; 95CI% [− 0.22; − 0.05]; *p* = 0.003). The quality of the matching was good, as shown in Fig. 2C and Additional file 8. This observation was confirmed in the sensitivity analysis, where a high serum HDL-C level was independently associated with lower odds of poor functional outcome (adjusted OR 0.60; 95% CI [0.40; 0.90]; *p* = 0.002). We observed a significant shift in the overall distribution of 90-day mRS scores in favour of the high HDL-C group over the low HDL-C group (adjusted OR 0.60; 95CI% [0.44; 0.81]; *p* = 0.001), as shown in Fig. 3.

### Association of HDL-C levels and neurological deficits at discharge

A high serum HDL-C level was associated with a 2.3-point lower NIHSS score at discharge compared to a low serum HDL-C level (ß = − 2.32; 95CI% [− 4.13; −0.53]; *p* = 0.01). The beneficial effect of higher serum HDL-C levels was not reproducible when additionally adjusting for reperfusion injury.

## Discussion

The main finding of this study is that in a prospective registry cohort of patients undergoing thrombectomy for acLVO, higher serum HDL-C levels reduced the odds of post-interventional ICH and were associated with better functional outcome at 90 days and less severe neurological deficits at the time of discharge. These observations could not be explained by cardiovascular risk profiles and conventional predictors of poor clinical outcome after thrombectomy. Whether this observation suggests a beneficial influence of HDL-C on the effects of reperfusion at the time of endovascular intervention requires further investigation.

The issue of reperfusion injury after thrombectomy has grown exponentially in recent years due to the rapid expansion of the indications for this treatment. This is due to an extended time window from symptom onset and a larger area of demarcated infarction that still allows the patient to be eligible for thrombectomy [16, 17]. As a result, the expansion of the indication has increased the possible amount of cumulative structural brain damage at the time point when reperfusion reaches ischaemic brain tissue. The potential clinical relevance of HDL-C-mediated protection against reperfusion injury is highlighted by a recent analysis of the prospective CIPPIS registry. This analysis showed that reperfusion injury was independently associated with poor functional outcome at 3 months in patients with successful recanalisation of acLVO [12]. Although there is considerable heterogeneity in the way cerebral reperfusion injury is defined, in our cohort we were able to demonstrate that patients with post-interventional ICH after thrombectomy for acLVO had 2.87-fold higher odds of poor functional outcome. We selected specific imaging indices of post-interventional ICH, including H1, H2, PH1, PH2, and class 3c haemorrhage according to the Heidelberg Bleeding Classification, as these represent the final stages of reperfusion-related blood–brain barrier damage and have therefore been proposed to be the key radiological features of reperfusion injury [9, 15].

Large randomised trials have evaluated the effects of candidate cerebroprotective agents, such as nerinetide and uric acid, on clinical outcomes in acLVO patients undergoing thrombectomy [18, 19]. Neither study provided sufficient evidence to legitimise widespread clinical use of the investigational agents tested. More recently, experimental strategies have been developed to specifically target and counteract reperfusion injury after thrombectomy. These approaches include ischaemic postconditioning and pharmacological attenuation of NMDA receptor-mediated excitotoxicity and free radical toxicity. They are currently being tested in clinical trials [20, 21]. It is important to recognise that the pathways by which HDL-C may protect reperfused brain tissue from injury are complex and probably include preservation of the integrity of the blood–brain barrier through activation of the scavenger receptor class B type I [11], restricted overexpression of adhesion molecules by endothelial cells [10], and attenuated neutrophil recruitment [22]. However, these observations are based on animal studies. Their applicability to human brain tissue is unclear. A recent study has translated evidence from animal research on the cardioprotective effects of HDL-C into clinical research. The AEGIS-II trial investigated the effects of treatment with apolipoprotein A-1, which is the major protein in HDL-C, in patients with myocardial infarction and multivessel coronary artery disease. This randomised controlled study did not demonstrate superiority over placebo in preventing major adverse cardiovascular events [23]. However, the cardioprotective effects of apolipoprotein A-1 are likely to be mediated by remodelling of intraluminal plaque formations in the coronary arteries, which may not translate into protection of the blood–brain barrier in the scenario of cerebral reperfusion. We focused our analysis on the haemorrhagic consequences of cerebral reperfusion injury and found consistent associations between higher HDL-C levels and reduced structural brain damage. Therefore, the potential protective effects of HDL-C may be strongest in preventing the final stages of cerebral reperfusion injury, when disruption of the blood–brain barrier leads to parenchymal haemorrhage.

### Strengths and limitations

Our study is subject to the limitations of a non-randomised design. However, we used propensity score matching to balance covariates between the two groups created with high and low serum HDL-C levels and we were able to reproduce our observations in sensitivity analyses using multivariable regression models. We did not assess rates of expansion of the ischaemic core and penumbra over time to capture possible ischaemic manifestations of reperfusion injury. However, we focused our study on haemorrhagic complications of reperfusion injury in order to clearly capture a terminal manifestation of the pathophysiological cascade leading up to blood–brain barrier disruption. Hence, we cannot comment on the temporal dynamics of the development of the cerebroprotective effects of HDL-C in specific phases of this cascade. Our imaging outcome of post-interventional ICH subsumes haemorrhagic transformation and parenchymal or subarachnoid haemorrhage. Parenchymal or subarachnoid haemorrhage seen after thrombectomy may also result from intraprocedural complications of thrombectomy, such as vessel injury or perforation, potentially diluting the composite outcome. However, the prevalence of this interventional complication is low ranging from 0.6 to 5.5% [6, 24]. It did not affect the robustness of the observations in our study. Reperfusion injury can manifest up to 72 h after thrombectomy [3]. We performed post-interventional follow-up CT within 24 h and may have missed cases of late-onset haemorrhagic reperfusion injury. The generalisability of the results of our study is limited by its monocentric design. A multicentre investigation of the effects of HDL-C on reperfusion injury after thrombectomy for acLVO is needed. Finally, the observed association between higher HDL-C levels and a favourable functional outcome at 90 days may be partly due to a long-term protective effect of HDL-C not related to reperfusion mechanisms. However, we also found an association between higher serum HDL-C levels and lower severity of neurological deficits at discharge that could not be reproduced when we additionally adjusted for post-interventional ICH. This observation may be consistent with an immediate beneficial effect of HDL-C on neurological outcome, mediated by reduced ICH after thrombectomy.

## Conclusions

In patients with acLVO, a high serum HDL-C level reduced the probability of ICH after thrombectomy and was associated with less neurological deficits at discharge and a favourable functional outcome at 90 days. These associations could not be explained by cardiovascular risk profiles and conventional risk factors for a poor clinical outcome after thrombectomy. A multicentre study is needed to investigate whether HDL-C may have a previously unrecognised influence on the integrity of the blood–brain barrier during reperfusion in humans, which may extend the role of HDL-C beyond traditional long-term cardiovascular protection.

## Supplementary Information

Additional file 1.

Additional file 2.

Additional file 3.

Additional file 4.

Additional file 5.

Additional file 6.

Additional file 7.

Additional file 8.

## Data Availability

The datasets used and/or analysed during the current study are available from the corresponding author on reasonable request.

## Abbreviations

AIS: Acute ischemic stroke
acLVO: Anterior circulation large vessel occlusion
ASPECTS: Alberta stroke program early CT score
ICH: Intracranial hemorrhage
IVT: Intravenous thrombolysis
mTICI: Modified thrombolysis in cerebral infarction
NIHSS: National institute of health stroke scale
mRS: Modified rankin scale
TOAST: Trial of org 10,172 in acute stroke treatment
