# Supplementary material for: Association of high-density lipoprotein cholesterol with reduced intracranial haemorrhage and favourable functional outcome after thrombectomy for ischaemic stroke: a propensity-matched analysis
Source: Neurol Res Pract. 2025 Mar 10;7(1):16. doi: 10.1186/s42466-025-00373-4 (PMC11921977; doi:10.1186/s42466-025-00373-4)
Supplement: Supplementary file 1 — Additional file 1. [file 42466_2025_373_MOESM1_ESM.pdf]

Additonal file 1: STROBE Checklist

	Item No.	Recommendation	Page No.	Relevant text from manuscript
**Title and abstract**	1	(*a*) Indicate the study's design with a commonly used term in the title or the abstract	1-3	"We included consecutive patients from our prospective anterior circulation large vessel occlusion (acLVO) registry who underwent thrombectomy between 01/2017 and 01/2023 at a tertiary stroke centre in Germany in a propensity score-matched analysis."
		(*b*) Provide in the abstract an informative and balanced summary of what was done and what was found	2-3	"In patients undergoing thrombectomy for acLVO, higher HDL-C levels were associated with a reduced probability of post-interventional ICH and a favourable functional outcome, providing a potential target for cerebroprotection."
**Introduction**				
Background/rationale	2	Explain the scientific background and rationale for the investigation being reported	3-4	"Radiologically confirmed haemorrhagic reperfusion injury occurs in up to one third of patients after thrombectomy with partial or complete reperfusion, worsening functional outcome."
Objectives	3	State specific objectives, including any prespecified hypotheses	4	"We tested the hypothesis that patients with higher serum HDL-C levels undergoing thrombectomy for acLVO would have a lower risk of intracranial haemorrhage (ICH) after the intervention than those with lower HDL-C levels."
Methods				
Study design	4	Present key elements of study design early in the paper	4-5	"…prospective endovascular treatment registry…retrospective cohort study."
Setting	5	Describe the setting, locations, and relevant dates, including periods of recruitment, exposure, follow-up, and data collection	4-5	"…at the tertiary stroke centre of the University Hospital Carl Gustav Carus in Dresden, Germany between 01/2017 and 01/2023."
Participants	6	(*a*) *Cohort study*—Give the eligibility criteria, and the sources and methods of selection of participants. Describe methods of follow-up	4-5	"We included all adult acLVO patients…who underwent thrombectomy…"
		(*b*) *Cohort study*—For matched studies, give matching criteria and number of exposed and unexposed<br>*Case-control study*—For matched studies, give matching criteria and the number of controls per case	6-7, Additional files 6-8	"The maximum allowed difference in propensity scores for matching (calliper value) was aimed at <0.2 (fraction of the standard deviation of the logit of the propensity score) to ensure high quality matching. Visual and

				analytical comparisons were performed to assess the quality of the matches.”
Variables	7	Clearly define all outcomes, exposures, predictors, potential confounders, and effect modifiers. Give diagnostic criteria, if applicable	6-7, Additional file 2	“Covariates were selected by multivariable logistic regression including clinically relevant covariates, namely age, sex, premorbid dependency, chronic diseases that may affect functional independence, baseline NIHSS score, Alberta Stroke Program Early CT Score (ASPECTS),...”
Data sources/ measurement	8*	For each variable of interest, give sources of data and details of methods of assessment (measurement). Describe comparability of assessment methods if there is more than one group	5-6, Additional files 2 and 4	“All brain scans were evaluated by board-certified neuroradiologists. Successful recanalisation was defined as an mTICI score of 2b or greater. Functional outcome was assessed by telephone interview 90 days after thrombectomy.”
Bias	9	Describe any efforts to address potential sources of bias	6-7	“In the sensitivity analysis, we performed multivariable lasso regression to assess the association between serum HDL-C levels and imaging indices of post-interventional ICH and favourable functional outcome, as well as early NIHSS score at discharge, adjusting for clinically relevant covariates. Residuals were tested for normality.”
Study size	10	Explain how the study size was arrived at	4-5, Figure 1	“We included all adult acLVO patients from our prospective endovascular treatment registry who underwent thrombectomy at the tertiary stroke centre of the University Hospital Carl Gustav Carus in Dresden, Germany between 01/2017 and 01/2023 in a retrospective cohort study.”

Quantitative variables	11	Explain how quantitative variables were handled in the analyses. If applicable, describe which groupings were chosen and why	6-7	“Independent continuous variables were tested for normality using descriptive and analytical criteria (Shapiro-Wilk test).“ “Patients were divided into two groups based on their serum HDL-C level, with the cut-off value set at the median HDL-C level of the entire study population.”
Statistical methods	12	(*a*) Describe all statistical methods, including those used to control for confounding	6-7	“Propensity score matching was performed for the main analysis. Patients were divided into two groups based on their serum HDL-C level, with the cut-off value set at the median HDL-C level of the entire study population. Accordingly, low HDL-C was defined as an HDL-C level < 1.15 mmol/L and high HDL-C ≥ 1.15

				mmol/L. Covariates were selected by multivariable logistic regression including clinically relevant covariates, namely age, sex, premorbid dependency, chronic diseases that may affect functional independence, baseline NIHSS score, Alberta Stroke Program Early CT Score (ASPECTS), occlusion site, concomitant extracranial carotid occlusion, mTICI score, emergency carotid stenting, thrombectomy, onset-to-recanalization time, arterial hypertension, HbA1c (%) and serum level of low-density lipoprotein cholesterol (LDL-C) (mg/dl). Propensity score matching was performed for all covariates that showed a statistically significant association with the grouping variable and the outcome on this regression model. Propensity scores were generated using logistic regression.”
		(*b*) Describe any methods used to examine subgroups and interactions	6-7	“Patients were divided into two groups based on their serum HDL-C level, with the cut-off value set at the median HDL-C level of the entire study population. Accordingly, low HDL-C was defined as an HDL-C level < 1.15 mmol/L and high HDL-C ≥ 1.15 mmol/L.”
		(*c*) Explain how missing data were addressed	6-7, Additional file 5	“Available case analysis was carried out.” “The number of missing registry data was low. Details are provided in Additional file 5.”
		(*d*) *Cohort study*—If applicable, explain how loss to follow-up was addressed	N/A	
		(e) Describe any sensitivity analyses	6-7	“In the sensitivity analysis, we performed multivariable lasso regression to assess the association between serum HDL-C levels and imaging indices of post-interventional ICH and favourable functional outcome, as well as early NIHSS score at discharge, adjusting for clinically relevant covariates.”
**Results**				
Participants	13*	(a) Report numbers of individuals at each stage of study—e.g. numbers potentially eligible, examined for eligibility, confirmed eligible, included in the study, completing follow-up, and analyzed	8-9, Figure 1	“Subject selection and reasons for exclusion are shown in the study flowchart (Figure 1).”
		(b) Give reasons for non-participation at each stage	Figure 1	See Figure 1
		(c) Consider use of a flow diagram	Figure 1	See Figure 1
Descriptive data	14*	(a) Give characteristics of study participants (e.g., demographic, clinical, social) and information on exposures and potential confounders	8-9, Table 1	“Demographic and clinical characteristics, vascular risk profiles and imaging characteristics are detailed in Table 1.”

		(b) Indicate number of participants with missing data for each variable of interest	8-9, Figure 1, Additional file 5	"The number of missing registry data was low. Details are provided in Additional file 5."
		(c) *Cohort study*—Summarize follow-up time (e.g., average and total amount)	Figure 1	See Figure 1
Outcome data	15*	*Cohort study*—Report numbers of outcome events or summary measures over time	8-9, Table 1, Table 2, Figure 3	"The median mRS score at 90 days was 3 [IQR 1-4] in the high serum HDL-C group and 4 [IQR 2-6] in the low serum HDL-C group."
Main results	16	(*a*) Give unadjusted estimates and, if applicable, confounder-adjusted estimates and their precision (eg, 95% confidence interval). Make clear which confounders were adjusted for and why they were included	8-9, Figure 3 Additional files 6-8	"The presence of post-interventional ICH on brain imaging was associated with a 14.8% increase in the probability of poor functional outcome (ß=0.15; 95CI% [0.06; 0.24]; p=0.001."
		(*b*) Report category boundaries when continuous variables were categorized	6-7	"Accordingly, low HDL-C was defined as an HDL-C level < 1.15 mmol/L and high HDL-C ≥ 1.15 mmol/L."
		(*c*) If relevant, consider translating estimates of relative risk into absolute risk for a meaningful time period	N/A	
Other analyses	17	Report other analyses done—e.g. analyses of subgroups and interactions, and sensitivity analyses	8-9, Figure 3	"We observed a significant shift in the overall distribution of 90-day mRS scores in favour of the high HDL-C group over the low HDL-C group (adjusted OR 0.60; 95CI% [0.44; 0.81]; p=0.001), as shown in Figure 3."
**Discussion**				
Key results	18	Summarize key results with reference to study objectives	10-13	"The main finding of this study is that in a prospective registry cohort of patients undergoing thrombectomy for acLVO, higher serum HDL-C levels reduced the odds of post-interventional ICH and were associated with better functional outcome at 90 days and less severe neurological deficits at the time of discharge. These observations could not be explained by cardiovascular risk profiles and conventional predictors of poor clinical outcome after thrombectomy. Whether this observation suggests a beneficial influence of HDL-C on the effects of reperfusion at the time of endovascular intervention requires further investigation."

Limitations	19	Discuss limitations of the study, taking into account sources of potential bias or imprecision. Discuss both direction and magnitude of any potential bias	12-13	"Our study is subject to the limitations of a non-randomised design."
Interpretation	20	Give a cautious overall interpretation of results considering objectives, limitations, multiplicity of analyses, results from similar studies, and other relevant evidence	13	"In patients with acLVO, a high serum HDL-C level reduced the probability of ICH after thrombectomy and was associated with less neurological deficits at discharge and a favourable functional outcome at 90 days. These associations could not be explained by cardiovascular risk profiles and conventional risk factors for a poor clinical outcome after thrombectomy. A multicentre study is needed to investigate whether HDL-C may have a previously unrecognised influence on the integrity of the blood-brain barrier during reperfusion in humans, which may extend the role of HDL-C beyond traditional long-term cardiovascular protection."
Generalizability	21	Discuss the generalizability (external validity) of the study results	12-13	"The generalisability of the results of our study is limited by its monocentric design. A multicentre investigation of the effects of HDL-C on reperfusion injury after thrombectomy for acLVO is needed."
**Other information**				
Funding	22	Give the source of funding and the role of the funders for the present study and, if applicable, for the original study on which the present article is based	15	"This study received no external funding. Open access funding covered by Project DEAL."

*Give information separately for cases and controls in case-control studies and, if applicable, for exposed and unexposed groups in cohort and cross-sectional studies.

Note: An Explanation and Elaboration article discusses each checklist item and gives methodological background and published examples of transparent reporting. The STROBE checklist is best used in conjunction with this article (freely available on the Web sites of PLoS Medicine at http://www.plosmedicine.org/, Annals of Internal Medicine at http://www.annals.org/, and Epidemiology at http://www.epidem.com/). Information on the STROBE Initiative is available at www.strobe-statement.org.
