# Supplementary material for: Association of high-density lipoprotein cholesterol with reduced intracranial haemorrhage and favourable functional outcome after thrombectomy for ischaemic stroke: a propensity-matched analysis
Source: Neurol Res Pract. 2025 Mar 10;7(1):16. doi: 10.1186/s42466-025-00373-4 (PMC11921977; doi:10.1186/s42466-025-00373-4)
Supplement: Supplementary file 2 — Additional file 2. [file 42466_2025_373_MOESM2_ESM.pdf]

Additional File 2 Definitions of patient characteristics

List of definitions

1. Occlusion sites

We included only patients with acLVO defined as complete occlusion of at least one large intracranial artery on computed tomographic angiography (CTA), i.e., the intracranial segment of the internal carotid artery (ICA), the M1 segment of the middle cerebral artery (MCA), the M2 segment of the MCA. This included combined occlusions, i.e., tandem occlusion, carotid T occlusion, carotid L occlusion, carotid I occlusion). We excluded patients with isolated anterior cerebral artery (ACA) occlusion because thrombectomy is less established in this territory. Posterior circulation occlusion was also excluded.

2. National Institutes of Health Stroke Scale at baseline

3. In community hospitals without neurological expertise, which serve as the spokes of our telestroke network, patients were assessed using the National Institutes of Health Stroke Scale (NIHSS) by a telestroke fellow at the coordinating tertiary stroke centre via videoconference. Local neurologists assessed drip and ship patients who were transferred from a partner hospital with a neurology department. After transfer or direct admission to the tertiary stroke centre, all patients were assessed by vascular neurologists. All patients were assessed for baseline NIHSS. In cases where the NIHSS score was not available but a detailed report of the neurological physical examination was available, the NIHSS score was determined post hoc by an investigator (AS). Mechanical ventilation with sedation was equivalent to a NIHSS score of 32 points.

4. Premorbid state

We considered patients to be dependent if regular assessment showed a continuing need for assistance in any dimension of activities of daily living [Supplementary ref. 1].

Supplementary ref. 1 Katz S (1983) Assessing self-maintenance: activities of daily living mobility, and instrumental activities of daily living. J Am Geriatr Soc 31:721–727. https://doi.org/10.1111/j.1532-5415.1983.tb03391.x

5. Chronic disease

- We defined a chronic disease as a condition that may affect functional independence. Diseases were classified according to the organ systems listed below, with one point awarded for each system:
- Cor/Vascular: coronary artery disease, history of myocardial infarction, high grade vitium, peripheral artery disease, non-compaction cardiomyopathy, history of carotid artery stenting
- Pulmonary: asthma, pulmonary hypertension, chronic obstructive pulmonary disease, chronic hypoventilation syndrome
- Renal: renal insufficiency
- Abdomen: chronic pancreatitis or hepatitis, sclerosing cholangitis, cholesterol crystal embolization, liver failure, previous Billroth I operation
- Other organ systems: neurodegenerative diseases, Churg-Strauss syndrome, rheumatoid arthritis, myasthenia gravis, hepatic steatosis, neurosarcoidosis, critical illness polyneuropathy, critical illness myopathy, polymyalgia rheumatica, systemic sclerosis, factor V Leiden, paranoid schizophrenia, infantile brain injury, muscular dystrophy, multiple sclerosis, morbid obesity
