# Supplementary material for: Association of high-density lipoprotein cholesterol with reduced intracranial haemorrhage and favourable functional outcome after thrombectomy for ischaemic stroke: a propensity-matched analysis
Source: Neurol Res Pract. 2025 Mar 10;7(1):16. doi: 10.1186/s42466-025-00373-4 (PMC11921977; doi:10.1186/s42466-025-00373-4)
Supplement: Supplementary file 3 — Additional file 3. [file 42466_2025_373_MOESM3_ESM.pdf]

Additional file 3 Thrombectomy Registry

Details of the Dresden Thrombectomy Registry

Our prospective observational thrombectomy registry includes patients admitted directly to our tertiary stroke centre. It also includes drip-and-ship transfers from eight partner hospitals with neurology departments but no or limited thrombectomy capacity, and 13 community hospitals without neurology departments, which act as the spokes of our hub-and-spoke telestroke network. Further details of our stroke network have been published elsewhere [Supplementary ref. 2].

Supplementary ref. 2 Simon E, Forghani M, Abramyuk A, Winzer S, Wojciechowski C, Pallesen LP, Siepmann T, Reichmann H, Puetz V, Barlinn K, Barlinn J (2021) Intravenous Thrombolysis by Telestroke in the 3- to 4.5-h Time Window. Front Neurol 12:756062. https://doi.org/10.3389/fneur.2021.756062
