# Supplementary material for: Association of high-density lipoprotein cholesterol with reduced intracranial haemorrhage and favourable functional outcome after thrombectomy for ischaemic stroke: a propensity-matched analysis
Source: Neurol Res Pract. 2025 Mar 10;7(1):16. doi: 10.1186/s42466-025-00373-4 (PMC11921977; doi:10.1186/s42466-025-00373-4)
Supplement: Supplementary file 4 — Additional file 4. [file 42466_2025_373_MOESM4_ESM.pdf]

Additional file 4 Baseline and study parameters with mode of acquisition

	Available in prospective database	Extracted retrospectively via chart review
Demographic characteristics	X	
Premorbid condition		X
Chronic disease		X
Cardiovascular risk factors		
Arterial hypertension	X	X
Diabetes mellitus	X	
HbA1c		X
LDL-C		X
HDL-C		X
Medication		X
Stroke characteristics	X	
NIHSS at baseline	X	X
Carotid T occlusion		X
Tandem occlusion		X
Interventions	X	
Sedative regimen	X	
Procedural times, min	X	
Procedural outcomes	X	
mTICI	X	X
Post-Interventional ICH		X

IQR, interquartile range; SD standard deviation; LDL, low density lipoprotein; HDL, high density IQR, interquartile range; SD, standard deviation; LDL, low-density lipoprotein; HDL, high-density lipoprotein; HbA1C, hemoglobin A1c; NIHSS, National Institutes of Health Stroke Scale; mRS, modified Rankin scale; ASPECTS, Alberta Stroke Program Early CT score; TOAST, Trial of Org 10172 in Acute Stroke Treatment; IVT, intravenous thrombolysis; mTICI, modified treatment in cerebral infarction score. Parameters of interest for our study that were not available in our registry were extracted by chart review by two independent investigators (AS, SS). In cases where catheter angiography was omitted, e.g. due to early recanalisation or insufficient mismatch on perfusion imaging at the mother ship clinic after telemedicine consultation and subsequent drip-and-ship transfer, two experienced neuroradiologists (DK, AC) assessed the mTICI score post hoc using CT angiography as previously described [Supplementary ref. 3]. Consensus was reached for ambiguous findings.

Supplementary ref. 3 Mair G, von Kummer R, Adami A, White PM, Adams ME, Yan B, Demchuk AM, Farrall AJ, Sellar RJ, Ramaswamy R, Mollison D, Boyd EV, Rodrigues MA, Samji K, Baird AJ, Cohen G, Sakka E, Palmer J, Perry D, Lindley R, Sandercock PA, Wardlaw JM, IST-3 Collaborative Group (2015) Observer reliability of CT angiography in the assessment of acute ischaemic stroke: data from the Third International Stroke Trial. Neuroradiology 57:1-9. https://doi.org/10.1007/s00234-014-1441-0
