# Supplementary material for: Association of high-density lipoprotein cholesterol with reduced intracranial haemorrhage and favourable functional outcome after thrombectomy for ischaemic stroke: a propensity-matched analysis
Source: Neurol Res Pract. 2025 Mar 10;7(1):16. doi: 10.1186/s42466-025-00373-4 (PMC11921977; doi:10.1186/s42466-025-00373-4)
Supplement: Supplementary file 5 — Additional file 5. [file 42466_2025_373_MOESM5_ESM.pdf]

Additional file 5 Missing data

Missing data in the prospective registry of patients undergoing thrombectomy

Parameter	Missing data (n, %)
Age	0, 0.00
Sex, female	0, 0.00
Arterial hypertension	2, 0.25
Diabetes mellitus	0, 0.00
Wake-up stroke	16, 1.98
NIHSS at discharge	5, 0.62
mRS at baseline	2, 0.25
mRS at discharge	5, 0.62
ASPECTS	4, 0.50
Occlusion site	0, 0.00
Leptomeningeal collaterals on DSA	8, 0.99
TOAST classification	3, 0.37
Interventions	0, 0.00
Sedative regimen	17, 2.11
Onset-to-needle	411, 50.93
Onset-to-groin	58, 7.19
Onset-to-recanalization	123, 15.24
mTICI	0, 0.00

NIHSS, National Institutes of Health Stroke Scale; mRS, modified Rankin scale; ASPECTS, Alberta Stroke Program Early CT score; DSA, digital subtraction angiography; TOAST, Trial of Org 10172 in Acute Stroke Treatment; IVT, intravenous thrombolysis; mTICI, modified treatment in cerebral infarction score.
