# Supplementary material for: Association of high-density lipoprotein cholesterol with reduced intracranial haemorrhage and favourable functional outcome after thrombectomy for ischaemic stroke: a propensity-matched analysis
Source: Neurol Res Pract. 2025 Mar 10;7(1):16. doi: 10.1186/s42466-025-00373-4 (PMC11921977; doi:10.1186/s42466-025-00373-4)
Supplement: Supplementary file 6 — Additional file 6. [file 42466_2025_373_MOESM6_ESM.pdf]

Additional file 6 Covariates, standardised differences and variance ratios from propensity score matching – Part A

Covariates, standardised differences and variance ratios from propensity score matching to assess the association between post-interventional ICH and functional outcome

Covariates	Standardized differences		Variance ratio	
	Raw	Matched	Raw	Matched
Age	-0.1277	-0.0661	0.8236	0.7925
HbA1c (%)	0.2176	0.0635	1.6203	1.1264
HDL (mg/dl)	-0.3108	0.0702	0.9714	1.0011
Premorbid status	-0.1705	-0.0398	0.8279	0.9582
NIHSS at baseline	0.1444	0.0249	0.8635	0.8473
Tandem occlusion	0.0933	-0.0548	1.2232	0.8934
ASPECTS	-0.5316	-0.0257	1.1474	1.0170
Intravenous Thrombolysis	-0.1176	0.0833	1.0189	0.9849
Onset-to-recanalization time, OTR (min)	0.2551	0.0138	0.8646	1.2535
Log(OTR) Log(min))	0.3411	0.0131	0.6918	0.9218
mTICI	-0.0978	0.0445	1.0194	0.9256

National Institutes of Health Stroke Scale; ASPECTS, Alberta Stroke Program Early CT score; hemoglobin A1c; HDL, low-density lipoprotein; mTICI, modified treatment in cerebral infarction score. Onset-to-recanalization time was included together with its Log term to improve matching results due to a skewed distribution.
