# Supplementary material for: Association of high-density lipoprotein cholesterol with reduced intracranial haemorrhage and favourable functional outcome after thrombectomy for ischaemic stroke: a propensity-matched analysis
Source: Neurol Res Pract. 2025 Mar 10;7(1):16. doi: 10.1186/s42466-025-00373-4 (PMC11921977; doi:10.1186/s42466-025-00373-4)
Supplement: Supplementary file 7 — Additional file 7. [file 42466_2025_373_MOESM7_ESM.pdf]

Additional file 7 Covariates, standardised differences and variance ratios from propensity score matching: Part B

Covariates, standardised differences and variance ratios from propensity score matching to assess the association between high HDL-C levels and post-interventional ICH

Covariates	Standardized differences		Variance ratio	
	Raw	Matched	Raw	Matched
Age	0.1313	-0.0286	1.0411	1.0724
Sex	-0.4809	0.0416	0.9925	0.9993
HbA1c (%)	-0.3024	-0.0632	0.5881	0.9932
$HbA1c^2$ ($\%^2$)	-0.2974	-0.0561	0.5772	1.0491
ASPECTS	0.0941	0.0102	0.9003	1.0693
Intravenous thrombolysis	0.0161	-0.0803	0.9974	1.0096
Onset-to-recanalization time, OTR (min)	-0.0835	0.0509	0.9300	0.9209
mTICI	0.0910	0.0509	0.9300	0.9209
Log(mTICI)	0.1110	-0.0406	0.6564	0.9536

ASPECTS, Alberta Stroke Program Early CT score; HbA1c, hemoglobin A1c; mTICI, modified treatment in cerebral infarction score; Onset-to-recanalization time was included together with its log term and HbA1c was included together with its quadratic term to improve matching results due to a skewed or non-linear distribution.
