# Supplementary material for: Association of high-density lipoprotein cholesterol with reduced intracranial haemorrhage and favourable functional outcome after thrombectomy for ischaemic stroke: a propensity-matched analysis
Source: Neurol Res Pract. 2025 Mar 10;7(1):16. doi: 10.1186/s42466-025-00373-4 (PMC11921977; doi:10.1186/s42466-025-00373-4)
Supplement: Supplementary file 8 — Additional file 8. [file 42466_2025_373_MOESM8_ESM.pdf]

Additional file 8 Covariates, standardised differences and variance ratios from propensity score matching: Part C

Covariates, standardised differences and variance ratios from propensity score matching to assess the association between high HDL-C levels and favourable functional outcome

Covariates	Standardized differences		Variance ratio	
	Raw	Matched	Raw	Matched
Age	0.1125	-0.6358	1.0438	1.0921
Sex	-0.4899	-0.0032	0.9941	1.0000
HbA1c (%)	-0.3001	-0.0328	0.6125	0.9404
Premorbid status	-0.0342	-0.0252	0.9643	0.9734
NIHSS at baseline	-0.0968	0.0817	0.9780	1.0683
Tandem occlusion	-0.1970	0.2442	0.6429	1.0589
ASPECTS	0.0742	0.0129	0.8809	0.9364
Intravenous thrombolysis	0.0233	0.0478	0.9963	0.9932
Onset-to-recanalization time (min)	-0.0868	-0.0730	0.9304	0.8954
Log(OTR) Log(min))	-0.0926	-0.0591	0.9990	0.8978

ASPECTS, Alberta Stroke Program Early CT score; HbA1c, hemoglobin A1c; Onset-to-recanalization time was included together with its log term to improve matching results due to a skewed distribution.
